# Supplementary material for: User preferences in multi-objective routes: The role of gradient visualization and personality measures
Source: PLoS One. 2025 Aug 6;20(8):e0329387. doi: 10.1371/journal.pone.0329387 (PMC12327598; doi:10.1371/journal.pone.0329387)
Supplement: S1 Appendix — Details of preprocessing and implementation to prepare route pairs (route A and route B) were provided. (PDF) [file pone.0329387.s001.pdf]

Method Details

We prepare candidate routes using multi-objective planning methods, which allows us to control them based on the most efficient path. Let us assume that an area is modeled as a graph $G = (V, E, \mathbf{l})$, where (V, E) represents an undirected graph structure with sets V and E of nodes and edges, and $\mathbf{l}$ includes multiple labels (e.g., l^{dist}, l^{alt}, and l^{rot} below) on V and E to represent multiple aspects of routes. All such additional information is now contained in $\mathbf{l}$ as (multi-dimensional) labels, and we assume that the vertex and edge labels could be well-defined and preprocessed.

In our scenario, a user attempts to walk from one vertex to another on G, with some walking route visualized on a map interface. Our system aims to recommend such routes following predesigned criteria. The objective values of path $p = \langle p_1, \ldots, p_L \rangle$ can be computed by summing the attached attributes. The travel cost is defined with $dist(p) := \sum_{1 \leq i \leq L-1} l^{dist}(p_i, p_{i+1})$, where $(p_i, p_{i+1}) \mapsto l^{dist}(p_i, p_i + 1) \in \mathbb{R}_{\geq 0}$ means the distance along with (p_i, p_{i+1}). If we are interested in gentler paths, we minimize $elev(p) := \sum_{1 \leq i \leq L-1} \max\{0, l^{alt}(p_{i+1}) - l^{alt}(p_i)\}$, where $l^{alt}(p_i)$ represents the altitude at vertex p_i.

To extract a manageable number of candidate routes from the vast pool of non-shortest routes, we adopt a multi-objective scenario that simultaneously minimizes the targeting metrics. In multi-objective settings like ours, we denote an m-dimensional score by $[d_1(p), \ldots, d_m(p)] \in \mathbb{R}^m$. Let p_1 and p_2 be two paths. A path p_1 is said to (Pareto) dominate another path p_2 if and only if (1) for any j $(1 \leq j \leq m)$ such that $d_j(p_1) \leq d_j(p_2)$ and (2) there exists j $(1 \leq j \leq m)$ such that $d_j(p_1) < d_j(p_2)$. The collection of non-dominated solutions is referred to as the Pareto front, and solutions belonging to this collection are termed Pareto optimal solutions.

In our experiments, a graph G is prepared as illustrated in Fig 1.

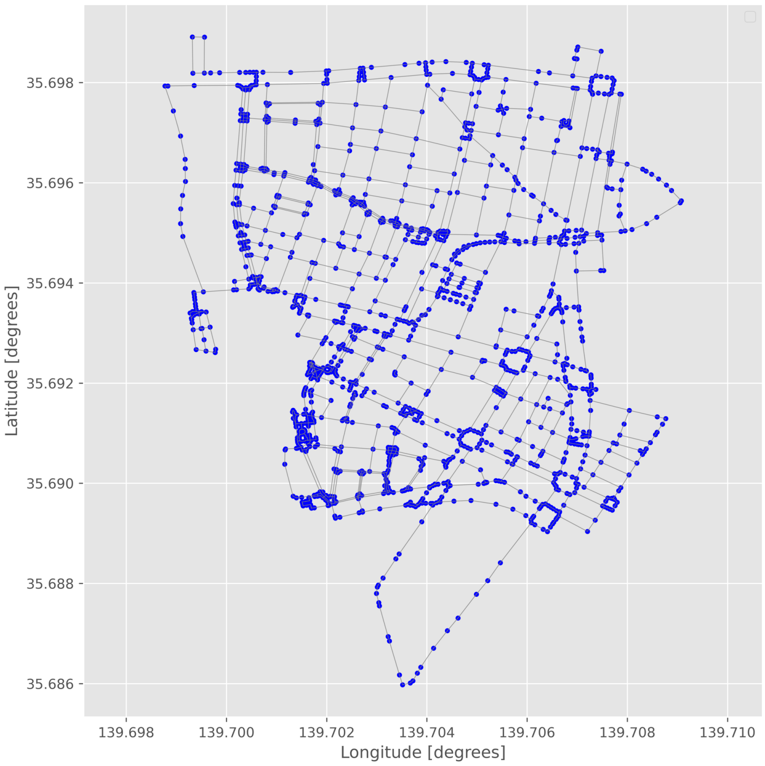

Figure 1: Graph in Shinjuku, Tokyo; both axes represent locations in degrees.

To investigate multi-objective trade-offs in experiments, $\mathbf{l} = [l^{dist}, l^{alt}]$ are attached and the objective value vectors are $[dist(p), alt(p)]$. BOA* algorithm inputs the start and goal vertices, and outputs the Pareto-front, as illustrated in Fig 2. Note that Route A is fixed and Route B is selected according to the Pareto-front and contexts.

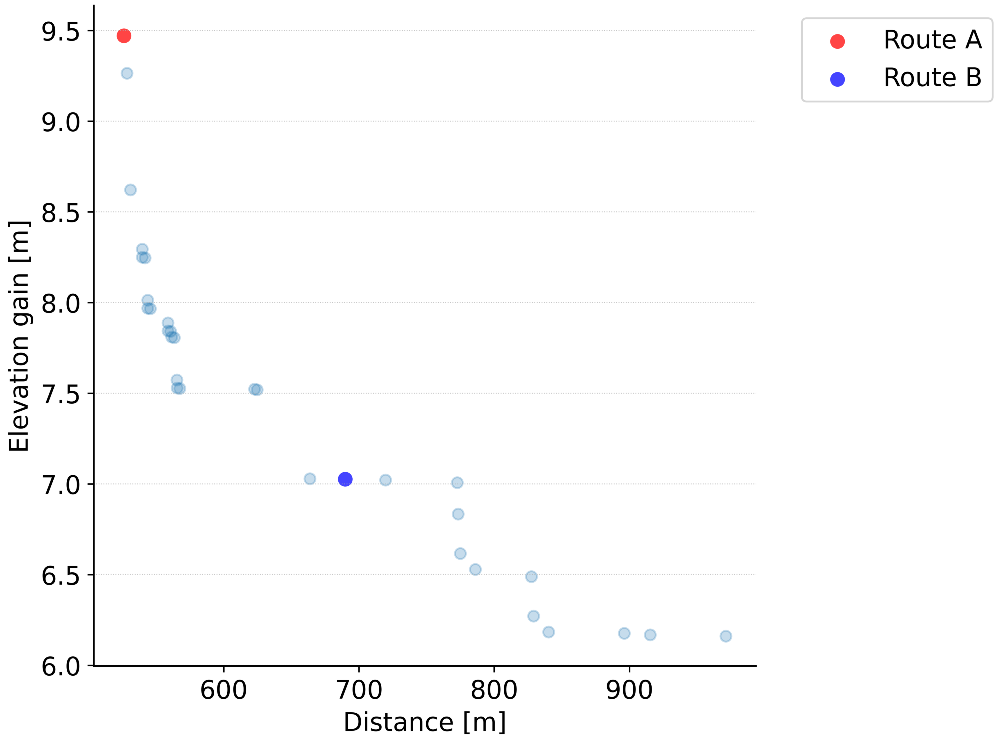

Figure 2: Pareto front and selected two routes (Route A and Route B).

From a route recommendation perspective, for two routes p_1 and p_2, if p_1 dominates p_2, the system does not need to take p_2; however, for two non-dominated routes p_1 and p_2, the system can present both to users for selection. Therefore, we can generate instances for participants to select routes using methods that compute the Pareto optimal routes.
