# Supplementary material for: User preferences in multi-objective routes: The role of gradient visualization and personality measures
Source: PLoS One. 2025 Aug 6;20(8):e0329387. doi: 10.1371/journal.pone.0329387 (PMC12327598; doi:10.1371/journal.pone.0329387)
Supplement: S2 Appendix — The questionnaire sheet was attached. (PDF) [file pone.0329387.s002.pdf]

Questionnaire for Personality Measures

This questionnaire was administered to all participants as part of the online survey.
It includes items for the Big Five personality traits, the Sensation Seeking Scale (SSS), and the Boredom-Alleviation (BA) subscale of the novelty-seeking measure.

Note: This PDF file was translated from Japanese to English for readability.

Big Five Personality Traits (10 items)

Participants were asked to indicate the extent to which they agree with each statement on a 7-point Likert scale:

(1) Disagree Strongly – (4) Neither Agree nor Disagree – (7) Agree Strongly

Item	Statement: I see myself as...
1	Extraverted, enthusiastic.
2	Critical, quarrelsome.
3	Dependable, self-disciplined.
4	Anxious, easily upset.
5	Open to new experiences, complex.
6	Reserved, quiet.
7	Sympathetic, warm.
8	Disorganized, careless.
9	Calm, emotionally stable.
10	Conventional, uncreative.

Sensation Seeking Scale (SSS; 8 items)

Participants were asked to respond to each item on a 5-point Likert scale:

(1) Strongly Disagree – (5) Strongly Agree

Item	Statement
1	I would like to explore strange places.
2	I get restless when I spend too much time at home.
3	I like to do frightening things.
4	I like wild parties.
5	I would like to take off on a trip with no pre-planned routes or timetables.
6	I prefer friends who are excitingly unpredictable.
7	I would like to try bungee jumping.
8	I would love to have new and exciting experiences, even if they are illegal.

Boredom-Alleviation (BA) Scale (3 items)

Participants responded to the following items on a 5-point Likert scale:

(1) Strongly Disagree – (5) Strongly Agree

Item	Statement
1	I want to travel to relieve boredom.
2	I have to go on vacation from time to time to avoid getting into a rut.
3	I like to travel because the same routine work bores me.
