# Supplementary material for: User preferences in multi-objective routes: The role of gradient visualization and personality measures
Source: PLoS One. 2025 Aug 6;20(8):e0329387. doi: 10.1371/journal.pone.0329387 (PMC12327598; doi:10.1371/journal.pone.0329387)
Supplement: Attachment — Submitted filename: point-to-point-response.pdf [file pone.0329387.s005.pdf]

Response to Reviewers

Point-to-Point Responses to Reviews for Manuscript PONE-D-25-17469: User preferences in multi-objective routes: the role of gradient visualization and personality measures

Keisuke Otaki[a] (otaki@mosk.tytlabs.co.jp) and Takayoshi Yoshimura[a] (yoshimura@mosk.tytlabs.co.jp)

[a] Toyota Central R&D Labs., Inc., Bunkyo-ku, Tokyo, Japan.

General Responses

We appreciate all the efforts of Editor-in-Chief, Associate Editor, and Reviewers for our manuscript. We have carefully revised our manuscript in response to each point raised. Please note that some sections (e.g., Introduction, parts of the Related Work) were comprehensively rewritten for clarity and structure. As such, not all edits are marked individually ('Revised Manuscript with Track Changes' highlights the important revision) in the revised submission to preserve readability.

Our point-to-point response document consists of three sections (Section #1, #2, and #3) for Editor and journal requirements (Section #1), Reviewer #1 (Section #2), and Reviewer #2 (Section #3). For visibility, the comments are also included from the decision letter (in this color), and each section includes point-to-point responses.

Section #1: Responses to Editor and Journal Requirements

Comment [J-1]

Please ensure that your manuscript meets PLOS ONE's style requirements, including those for file naming. The PLOS ONE style templates can be found at

- https://journals.plos.org/plosone/s/file?id=wjVg/PLOSOne_formatting_sample_main_body.pdf and
- https://journals.plos.org/plosone/s/file?id=ba62/PLOSOne_formatting_sample_title_authors_affiliations.pdf

Response to [J-1]

Author response and action. We have carefully checked and revised the manuscript following the instructions. We reviewed the PLOS ONE style guide and revised the manuscript accordingly. This includes the following updates.

- Formatting of the title page, author affiliations, section headings, and figure/table callouts.
- Consistent reference formatting based on the journal's specifications.
- Ensuring that all submitted files comply with file naming and layout requirements.

Comment [J-2]

We note that your Data Availability Statement is currently as follows: [All relevant data are within the manuscript and its Supporting Information files.]

Please confirm at this time whether or not your submission contains all raw data required to replicate the results of your study. Authors must share the "minimal data set" for their submission. PLOS defines the minimal data set to consist of the data required to replicate all study findings reported in the article, as well as related metadata and methods (https://journals.plos.org/plosone/s/data-availability#loc-minimal-data-set-definitio
For example, authors should submit the following data:

- The values behind the means, standard deviations and other measures reported;
- The values used to build graphs;
- The points extracted from images for analysis.

Authors do not need to submit their entire data set if only a portion of the data was used in the reported study. If your submission does not contain these data, please either upload them as Supporting Information files or deposit them to a stable, public repository and provide us with the relevant URLs, DOIs, or accession numbers. For a list of recommended repositories, please see https://journals.plos.org/plosone/s/recommended-repositories.

If there are ethical or legal restrictions on sharing a de-identified data set, please explain them in detail (e.g., data contain potentially sensitive information, data are owned by a third-party organization, etc.) and who has imposed them (e.g., an ethics committee). Please also provide contact information for a data access committee, ethics committee, or other institutional body to which data requests may be sent. If data are owned by a third party, please indicate how others may request data access.

Response to [J-2]

Author response and action. We have carefully checked and revised the manuscript following the instructions. In addition to the previously included raw data, we revised **S3 Appendix. Data**, which includes:

- Raw route-choice records per participant with aggregated statistics.
- Python plotting scripts used to generate figures in the Results section.

This update ensures full compliance with PLOS ONE's definition of a minimal dataset and supports reproducibility.

Comment [J-3]

Your ethics statement should only appear in the Methods section of your manuscript. If your ethics statement is written in any section besides the Methods, please move it to the Methods section and delete it from any other section. Please ensure that your ethics statement is included in your manuscript, as the ethics statement entered into the online submission form will not be published alongside your manuscript.

Response to [J-3]

Author response and action. We have carefully revised the manuscript following the instructions. The ethics statement is now included exclusively in the **Materials and Methods** → **Online Survey Procedure** subsection. We also ensured that the ethics statement entered in the online submission form matches the content in the manuscript.

Comment [J-4]

We note that Figures 1B, 2A, 2B, 2C in your submission contain [map/satellite] images which may be copyrighted. All PLOS content is published under the Creative Commons Attribution License (CC BY 4.0), which means that the manuscript, images, and Supporting Information files will be freely available online, and any third party is permitted to access, download, copy, distribute, and use these materials in any way, even commercially, with proper attribution.

Response to [J-4]

Author response and action. Thank you for pointing out this important issue. We have completely revised the relevant figures to eliminate any licensing concerns:

- Figs 1(b), 2(a), 2(b), and 2(c) previously contained Maps tiles (@Mapbox, @OpenStreetMap). These have been **removed** and **revised** in revision.
- Old Fig 1 has been replaced with a new Fig 1: conceptual diagram showing the experimental pipeline and participant assignment.
- The revised Fig 2 presents schematic visualizations. *These maps are based on Digital Japan Basic Maps with English labels, published by Geospatial Information Authority of Japan, whose license PDL 1.0 (Public Data License, Version 1.0), compatible with CC BY 4.0.*

All figures in the revised manuscript now fully comply with the CC BY 4.0 license.

Section #2: Point-to-Point responses to Reviewer #1

Comment [R1-1] on abstract

The abstract suffers from verbosity, vague terminology, and insufficient quantitative detail, reducing its conciseness and scientific rigor. The main problems are as follows:

- Verbose Language: Phrases like "we develop our framework to investigate" and repeated use of "our" (e.g., "our framework," "our survey") make the text less concise.
- Vague Terminology: Terms such as "multi-objective navigation," "gradient information," and "route-choice phenomena" are not clearly defined, potentially confusing readers.
- Lack of Specificity: Statements like "using modern multi-objective planning algorithms and crowdsourcing services" are overly broad, lacking details about the specific methods or technologies used.
- Insufficient Quantitative Detail: Claims such as "modestly encourages the selection of non-shortest and gentler routes" and "more pronounced preference" lack supporting data (e.g., percentages, statistical significance). The statement "route selection was also influenced by travel distance" is vague about the direction or magnitude of the effect.
- Unclear Relevance: Terms like "Big Five and Sensation Seeking dimensions" are standard in psychology but may confuse non-experts without context linking them to route choice.

Response to [R1-1]

Author response and action. Thank you for this insightful critique. Related to [R2-4], we have revised the Abstract as follows:

- Replaced general phrases like "we develop our framework" with more concise, neutral expressions.
- Clarified the use of "multi-objective" by referring explicitly to trade-offs between "slope and distance", consistent with the main text.
- Added "quantitative results", including participant size ($n = 91$), group mean comparisons (e.g., mean = 3.89 versus 4.29), and significance levels (e.g., $p < 0.01$).
- Simplified psychological terms by linking "Big Five" and "Sensation Seeking" directly to route choice behavior.

These revisions improve the clarity, precision, and informativeness of the Abstract for both specialist and general readers.

Comment [R1-2] on introduction

The introduction provides a foundation for navigation research and research questions but is undermined by unclear content, loose structure, insufficient rigor, redundant language, imprecise terminology, and unclear positioning, reducing its academic persuasiveness and readability. The following changes are recommended:

- Overly Detailed Example: Example 1 includes excessive specifics (e.g., path numbers, colors), diverting focus from research objectives and failing to link clearly to the study's goals.
- Weak Justification of Research Gap: The claim of "a significant gap" in understanding route evaluation lacks robust evidence, with only a few studies cited to support the link between personality traits and route choice, undermining the study's necessity.
- Redundant and Subjective Language: Repeated use of "we" (e.g., "we develop a framework") is overly subjective and detracts from the objective academic tone.

Response to [R1-2]

Author response and action. We appreciate the suggestions to improve structure and clarity. In response, we updated the manuscript as follows.

- The original "Example 1" was **removed** to reduce distraction and replaced by a concise route-choice scenario.
- We added a new paragraph that more clearly states the **research gap**, emphasizing the limited prior work linking route choice to **personality traits**.
- Subjective expressions (e.g., "we develop...") were rewritten using objective language.
- The Introduction now follows a clear structure: **Background** → **Related Work** → **Research Gap** → **Research Questions (RQ1–RQ3)**.

These changes improve flow and positioning while maintaining relevance.

Comment [R1-3] on related research

The related research section requires comprehensive refinement in content clarity, logical structure, scientific rigor, language expression, and terminology to enhance its academic value and readability. The following changes are recommended:

- Unclear Subsection Focus: Subsections (e.g., preparing navigation routes, personality in navigation, visualization and explanations) lack clear focus and logical connections, failing to align with the introduction's research questions (RQ1-RQ3).
- Insufficient Scientific Rigor: Key claims lack literature support, and speculative statements (e.g., "we can hypothesize that walking time information would be valuable") weaken credibility.
- Redundant and Inconsistent Language: Frequent use of phrases like "we can" or "for example" and inconsistent terminology (e.g., "personality measures" vs. other terms) reduce clarity.
- Vague Terminology: Terms like "crowdsourcing" and "multi-objective algorithms" are overly broad, lacking precise descriptions (e.g., "user-generated route data", "Pareto-based route optimization").

Response to [R1-3]

Author response and action. Thank you for your detailed feedback. We substantially revised the **Related Research** section to improve clarity, logical alignment, and academic rigor:

- The section is now organized around the three core research questions (RQ1–RQ3), with dedicated subsections on **multi-objective routing**, **personality in navigation**, and **visualization/explainability**.
- We replaced vague terms with precise technical descriptions: "Multi-objective algorithms" to "BOA* algorithm used to compute Pareto-optimal routes" and "Crowdsourcing" to "Online participant recruitment via CrowdWorks".
- Language was tightened, avoiding redundant phrases like "we can" and ensuring consistent use of "personality measures".
- We added recent references from 2022 until 2024 to strengthen the literature foundation.

Comment [R1-4] on results

The results section requires comprehensive refinement in content clarity, analytical logic, scientific rigor, language expression, and data presentation to enhance its academic rigor and readability. The following changes are recommended:

- Weak Analytical Logic: The analysis does not clearly address research questions (RQ1-RQ3), relying heavily on data descriptions (e.g., means, statistical tests) without qualitative interpretation of trends or implications.
- Insufficient Scientific Rigor: Key statistical details (e.g., effect sizes, confidence intervals) are missing, and data filtering criteria (e.g., "valid participants") are unclear.
- Unclear Personality Trait Analysis: The practical meaning of Sensation Seeking Scale (SSS) subdimensions (e.g., TAS, Dis) and their relevance to route choice are not explained beyond statistical differences.
- Redundant and Inconsistent Language: Phrases like "in the following, we..." are redundant, and terms like "gentler gradient" or "gradual route" are used inconsistently.
- Suboptimal Data Presentation: Direct references to figures (e.g., "Figure 4a shows...") are overused, and figure annotations are not self-explanatory, limiting accessibility for readers without visuals.
- The discussion is well-structured, but it lacks depth, specificity, and clarity in some areas, limiting its persuasiveness.

Response to [R1-4]

Author response and action. We revised the Results and Discussion sections to address scientific rigor and improve analytical depth:

- **Data Filtering**: The definition of "valid participants" was clarified in the Methods. Only participants who correctly answered two dummy questions were included ($n = 91$), as shown in Table 4.
- **Statistical Reporting**: All statistical comparisons include exact *p*values and group means. Figs 6–9 display means and standard errors.
- **Effect Sizes and Confidence Intervals**: In the revised manuscript, we added Cohen's d and 95% confidence intervals for key psychological comparisons (GENTLER_G vs. SHORTER_G). These were calculated from parametric estimates (means, pooled SD) even though non-parametric tests (Mann–Whitney U) were used for significance testing. This dual reporting is explained in the Discussion and follows common practice in behavioral research.
- **Psychological Interpretation**: The behavioral significance of each **SSS subdimension** (ES, BS, TAS, Dis) is now discussed in the Results and further interpreted in the Discussion, drawing from related work in psychology and tourism studies.
- **Figure Quality**: Figures were redesigned with clear English labels, consistent styling, and improved legends.

We believe these changes substantially improve the precision, interpretability, and scientific robustness of our findings.

Comment [R1-5] on discussions

The discussion is well-structured, but it lacks depth, specificity, and clarity in some areas, limiting its persuasiveness. The following changes are recommended:

- Superficial Result Interpretation: Responses to research questions (RQ1-RQ3) are clear but lack depth. For example, the conjecture about the lack of significant impact from gradient visualization (lines 406-407) lacks theoretical or data support.
- Brief Comparison to Prior Work: Comparisons with other studies (lines 423-441) are limited, mentioning only pedestrian-driver differences and personality measures, without highlighting the study's unique contributions.
- Incomplete Limitations Analysis: Limitations (e.g., small sample size, suboptimal map visualization, regional focus, online survey constraints) are identified but not analyzed for their specific impacts on results (e.g., effect on statistical significance).
- Broad Future Directions: Suggestions like field experiments and expanding route attributes are vague, lacking specific experimental designs or methods.
- Verbose and Vague Language: Sentences like "we can start by clustering users according to these scores when recommending non-shortest routes" (line 421) are verbose and lack precision.

Response to [R1-5]

Author response and action. We appreciate the detailed feedback comments. We revised the Discussion section to provide greater depth and specificity:

- Each research question (RQ1–RQ3) is now addressed in a bullet-point summary at the start of the section.
- We strengthened comparisons to prior work, especially regarding personality and mobility (e.g., Albert et al. 2011; Bekhor et al. 2014).
- Each limitation (sample size, online-only setting, regional scope) is explicitly linked to its possible effect on findings.
- Future work is specified in terms of:
  - Field experiments.
  - Multi-factor route modeling (e.g., safety, scenery, sidewalk quality).
  - Adaptive user interfaces informed by psychological profiles.

We also rephrased vague language and clarified our interpretive conjectures by referencing theoretical concepts. These changes improve the depth and applicability of our conclusions.

Comment [R1-6] on figures

The figures are inadequately designed, overly casual, and fail to meet scientific visualization standards due to unreasonable layouts, non-standard plotting, and missing or unclear annotations. For example,

- Figure 1a: The legend obscures substantial content, reducing clarity.
- Figure 1b: Lacks a legend and uses non-standard English expressions.
- Figures 2a and 2b: The maps are not in standard English, but in Japanese, limiting accessibility.
- Figure 6d: Lacks a legend, reducing interpretability.

Similar problems exist in other diagrams and full verification is recommended.

Response to [R1-6]

Author response and action. We completely redesigned all figures to meet scientific and visual standards:

- Figs 1a/b was replaced by a conceptual pipeline diagram illustrating the experimental process and route generation.
- Figs 2a-c was redrawn with schematic route visuals; all map tiles and Japanese labels were removed. English annotations are now used throughout. *These maps are based on Digital Japan Basic Maps with English labels, published by Geospatial Information Authority of Japan, whose license PDL 1.0 (Public Data License, Version 1.0), compatible with CC BY 4.0.*
- Fig 6d (now Fig 7) includes clearly labeled axes, data points, and a legend.
- All figures were reformatted with consistent fonts, improved layout, and properly positioned legends (e.g., Fig 5).
- Licensing issues were resolved: all visuals now comply with CC BY 4.0.

We believe these revisions bring the figures up to a high standard of scientific communication and accessibility.

Section #3: Point-to-Point responses to Reviewer #2

Comment [R2-1] on walking time differences

The walking time difference between Routes A and B is only one minute, which raises questions about whether such a marginal difference is sufficient to elicit meaningful user preference shifts. The authors should discuss the generalizability of their findings. Specifically, how representative are these two paths in broader urban contexts? Additional justification is needed to support the external validity and practical implications of the experimental design.

Response to [R2-1]

Author response and action. Thank you for this valuable comment. We clarified this issue in the revised **Discussion → Limitations** section as well.

Although the walking time difference between Route A and B was small in absolute terms, the relative difference in slope and effort was substantial–especially in long-distance cases. These scenarios reflect realistic trade-offs in urban pedestrian navigation, where short travel time differences often coexist with large physical or environmental constraints (e.g., stairs, steep gradients, or detours).

We also emphasize that consistent effects were observed across multiple route pairs and participant clusters, supporting robustness. Future work will test generalizability across more diverse urban and non-urban settings, as now described in the Limitations section.

Comment [R2-2] on multi-objective design

While the study discusses trade-offs between path distance and slope, it is unclear whether a formal multi-objective optimization framework was applied. If only two fixed paths (A and B) were compared without a systematic modeling of user preferences or Pareto front evaluation, describing the framework as "multi-objective" may be an overstatement. The authors are encouraged to clarify whether this is a conceptual framing or supported by a multi-objective algorithmic design.

Response to [R2-2]

Author response and action. We appreciate this important question regarding our core framework. We clarified our methodology in the **Materials and Methods → Multi-objective route generation** subsection and in **S1 Appendix**.

Our route generation relies on the BOA* algorithm, which efficiently computes Pareto-optimal routes with respect to both travel distance and cumulative elevation gain. Route A was selected as the shortest path, while Route B was selected from the Pareto set to reduce slope within a constrained detour (e.g., +10%, +30%). Thus, the route comparisons were derived from a systematic multi-objective search, not arbitrarily chosen, and are consistent with formal definitions of multi-objective optimization.

Comment [R2-3] on figures

Ensure all figures use English labels, and axes are clearly marked with units where applicable. Improve the visual clarity and design aesthetics of the graphs to enhance readability and data comprehension.

Response to [R2-3]

Author response and action. In response to this and related points ([J4], [R1-6]), we revised all figures to ensure scientific clarity and accessibility:

- All axis labels, annotations, and legends are now in English.
- Visual designs were standardized with improved typography, layout, and color schemes.
- Map tiles in Figure 2 were removed due to licensing, and schematic annotations were provided instead. Note that *maps are based on Digital Japan Basic Maps with English labels, published by Geospatial Information Authority of Japan, whose license PDL 1.0 (Public Data License, Version 1.0), compatible with CC BY 4.0.*

These revisions improve visual clarity and adhere to licensing and publication standards.

Comment [R2-4] on abstract

The method description in the abstract is too detailed; please condense it. Add key findings and outcomes to make the abstract more informative and balanced.

Response to [R2-4]

Author response and action. We thank for the suggestions to make abstract more informative and balance. We revised the Abstract to strike a better balance between method and findings:

- The methodological description is now condensed into a single sentence.
- Key **quantitative results** were added, including group means (e.g., 3.89 versus 4.29), significance levels ($p < 0.01$), and sample size ($n = 91$).
- Personality findings were reformulated to explicitly connect Sensation Seeking scores to non-shortest route preference.

These changes enhance the clarity and informativeness of the Abstract.

Comment [R2-5] on introduction

The introduction should be completely rewritten to follow standard academic structure: background, literature review, research objectives, and research gap. Do not include research findings in the Introduction section; such content should appear in the Results or Conclusion. Figure 1 should include a clear legend explaining the routes. Cite more recent studies from the last three years to strengthen the literature context and highlight the manuscript's contribution.

Response to [R2-5]

Author response and action. We appreciated your insightful suggestions. The Introduction has been thoroughly revised to follow a conventional academic structure:

- Background → Related work → Research gap → Research questions (RQ1-RQ3).
- We removed early findings (e.g., former Example 1) and subjective expressions.
- We also incorporated more recent studies to strengthen the literature base and highlight our contributions (e.g., [1, 2, 3]).

Comment [R2-6] on related research

The logic and flow of this section are unclear. Reorganize the content to present a coherent review of existing studies. Emphasize literature synthesis and critique rather than introducing this study's content. Statements directly describing the present research should be rephrased and placed toward the end of this section, for example: "Beyond these studies, we focus on collecting route-level preference data to address this gap...".

Response to [R2-6]

Author response and action. Thank you for the constructive feedback. We reorganized the **Related Research** section to align explicitly with our three research questions:

- Multi-objective route generation (RQ1)
- Psychological traits and decision-making (RQ3)
- Visualization and route explainability (RQ2)

This structure improves coherence and separates prior work from our original contributions.

Comment [R2-7] on methods and materials

The methodology is difficult to follow. A flowchart is recommended to visually present the research process. The description on Page 5 — "Fig. 1 conceptually illustrates the trade-off between route distance and gradient..." — lacks clarity. Please elaborate further. Justify why Routes A and B were selected. What are the environmental or physical characteristics surrounding these paths? The questionnaire should be included in the Appendix to enhance transparency and reproducibility.

Response to [R2-7]

Author response and action. We have revised the methodology section to improve clarity and reproducibility:

- Figure 1 was replaced with a schematic pipeline diagram illustrating the experimental flow.
- Route selection procedures are now more clearly described in **Materials and Methods** → **Multi-objective route generation** and **S1 Appendix**.
- The criteria for selecting Route A (shortest) and Route B (low gradient, limited detour) are explicitly justified (see lines 206–214).
- The full **questionnaire** has been added to **S2 Appendix**, including Big Five, SSS, and BA scales.

Comment [R2-8] on results

Some inconsistencies are noted between data description and figures. For example, "Figs. 3a and 3b show the histograms of the entered ages and gender of all participants and valid participants," yet Figure 3 does not clearly reflect this. The number "546" is mentioned without adequate explanation. Clarify its relevance to the figures or study design.

Response to [R2-8]

Author response and action. We addressed the inconsistencies in the Results section:

- Figure callouts (e.g., "Figs. 3(a) and 3(b)") are corrected and now match the updated figures.
- The "546" value is explicitly explained as follows:

> Finally, we obtained $(34+35+22)\times 6 = 91\times 6 = 546$ pairwise comparison data, where 91 represents the total number of valid participants.

This clarifies the data structure and its relevance to the figures: all figure callouts, related to revisions of our figures corresponding to [R2-3] and [R1-6].

Comment [R2-9] on conclusion

The conclusion should emphasize key findings, contributions, and potential applications. Avoid reiterating research motivations or background explanations in this section.

Response to [R2-9]

Author response and action. We greatly appreciate this comment. The **Conclusion** section was revised to avoid repetition and to better emphasize the contributions:

- Summary of empirical findings related to RQ1-RQ3.
- The role of personality traits (especially SSS) in shaping pedestrian route choices.
- Implications for the design of **personalized navigation systems**, with emphasis on adaptive UIs.

Redundant background material was removed for clarity and conciseness.

Comment [R2-10] on literature

You may find interest in the following reference for complementary insights.

Response to [R2-10]

Author response and action. Thank you for suggesting related studies. Our study on navigation and personality is obviously related to those studies in multi-dimensional routes in environment and smart city perspectives. We have revised our manuscript to highlight the wide application area of route-related studies in our revised manuscript.

References

Alexander Eggerth, Javier Argota Sánchez-Vaquerizo, Dirk Helbing, and Sachit Mahajan. Democratizing urban mobility through an open-source, multi-criteria route recommendation system. In *Proc. of the 18th ACM RecSys (RecSys 2024)*, pages 1073–1078, 2024.

José R Lozano-Pinilla and Cristina Vicente-Chicote. Parapp pedestrian-aware routing app. In *International Workshop on Gerontechnology*, pages 47–52. Springer, 2022.

Marina Torres, David A Pelta, and José L Verdegay. Proa: An intelligent multi-criteria personalized route assistant. *Engineering Applications of Artificial Intelligence*, 72:162–169, 2018.
