# Supplementary material for: User preferences in multi-objective routes: The role of gradient visualization and personality measures
Source: PLoS One. 2025 Aug 6;20(8):e0329387. doi: 10.1371/journal.pone.0329387 (PMC12327598; doi:10.1371/journal.pone.0329387)
Supplement: Attachment — Submitted filename: point-to-point-response_auresp_2.pdf [file pone.0329387.s007.pdf]

Response to Reviewers

Point-to-Point Responses to Reviews for Manuscript PONE-D-25-17469R1: User preferences in multi-objective routes: the role of gradient visualization and personality measures

Keisuke Otaki[a] (otaki@mosk.tytlabs.co.jp) and Takayoshi Yoshimura[a] (yoshimura@mosk.tytlabs.co.jp)

[a] Toyota Central R&D Labs., Inc., Bunkyo-ku, Tokyo, Japan.

General Responses

We appreciate all the efforts of Editor-in-Chief, Associate Editor, and Reviewers for our manuscript. We have carefully revised our manuscript in response to each point raised, particularly from our revised version (PONE-D-25-17469R1) based on our original submission (PONE-D-25-17469).

Our point-to-point response document consists of three sections (Section #1, #2, and #3) for pointed journal requirements (Section #1), Reviewer #1 (Section #2), and Reviewer #2 (Section #3). For visibility, the comments are also included from the decision letter (in this color), and each section includes point-to-point responses.

Section #1: Responses to Journal Requirements

Comment [J-1]

Please review your reference list to ensure that it is complete and correct. If you have cited papers that have been retracted, please include the rationale for doing so in the manuscript text, or remove these references and replace them with relevant current references. Any changes to the reference list should be mentioned in the rebuttal letter that accompanies your revised manuscript. If you need to cite a retracted article, indicate the article's retracted status in the References list and also include a citation and full reference for the retraction notice.

Response to [J-1]

Author response and action. We appreciate your notices. We have reviewed all cited works and found no retracted publications in our reference list. Thus, in the revised manuscript (i.e., 'Revised Manuscript with Track Changes'), we did not mark and mentioned this point on this revision round.

Section #2: Point-to-Point responses to Reviewer #1

General Comment from Reviewer #1

Thank you for submitting the revised manuscript. I greatly appreciate the effort and dedication you have invested in addressing the previous reviewer comments and improving the manuscript. The revisions demonstrate a commendable commitment to enhancing the clarity and scientific rigor of your work, and the study's contributions to the field are evident.

However, I must note that several issues regarding non-standardized figure formatting persist in the revised figures, which continue to impact the manuscript's overall presentation and readability. Specifically:

- Inconsistent Figure Formatting: While some figures have been improved, there remain inconsistencies in the style and formatting across figures. For example, the use of different line styles, marker sizes, or color schemes without clear justification creates a disjointed visual experience. I recommend adopting a uniform style guide for all figures to ensure consistency.
- Incomplete Axis Labeling: Certain figures still lack complete axis labels or units, which is critical for accurate data interpretation. For instance, units for numerical values or clear descriptors for categorical axes are missing in some cases. Please ensure that all axes are fully labeled with appropriate units and descriptions.
- Suboptimal Legend Design: The legends in several figures could be further refined. In some instances, legends are either overly crowded or not sufficiently descriptive, making it challenging to interpret the data quickly. I suggest simplifying and standardizing legend entries to improve clarity and accessibility.

Recommendation: These non-standardized plotting issues, while not detracting from the scientific merit of the study, significantly affect the professional presentation of the manuscript. I strongly recommend a thorough review and revision of all figures to align with the journal's graphical standards (e.g., PLOS ONE's figure guidelines). Utilizing professional visualization tools (e.g., Adobe Illustrator, R, or Python's Matplotlib with a consistent style configuration) could help achieve a polished and cohesive set of figures.

Response to Reviewer #1

Author response and action. We are happy to hearing from your confirmation on our revision conducted in PONE-D-25-17469R1. We thank for your comments and suggestions in the previous round, which contribute to improve our manuscript.

For further clarification, we again appreciate your insightful suggestions to make the presentation more consistent. To resolve the remaining issues, we would like to clarify and answer them in a point-to-point manner below.

As our general response, we have revised and prepared all main figure (Fig.3 - Fig.9) by Python with Matplotlib, as attached in our supplementary material. Our revision has clarified the inconsistent figure formatting issues, updated axis labeling and legend design as consistent as possible. Note that Maps in Fig.2 were designed by image editors with python scripts to present the situation of our online surveys using digital maps (see e.g., [1]); thus they are a bit different from matplotlib-based figures.

Comment [R1-1]: Absence of Legend in Figure 9

Figure 9 lacks a legend, rendering it impossible for readers to accurately interpret the elements depicted. This omission severely compromises the figure's readability and scientific rigor. I strongly recommend adding a clear and concise legend to Figure 9, ensuring that it corresponds directly to the figure's content.

Response to [R1-1]

Author response and action. We appreciate the suggestions to improve the result presentation in Figure 9. In our revised figure, the design and legend has been updated as consistent and precise as possible. Our revision could be helpful for readers to understand the findings illustrated in Figure 9.

Comment [R1-2]: Missing Units for Latitude and Longitude in Figure 10

The latitude and longitude axes in Figure 10 do not specify units (e.g., degrees, °), which violates standard scientific plotting conventions and risks ambiguity in data interpretation. I urge you to explicitly label the units for these axes and verify that similar issues do not affect other figures.

Response to [R1-2]

We appreciate the suggestions to improve our Figure 10. In our revised figure, the unit is explicitly given, the content and caption in appendix has been updated for clarity.

Comment [R1-3]: Inconsistent Placement of Legends

The placement of legends across multiple figures appears arbitrary, with some positioned within the figure and others outside, in varying locations. This inconsistency creates a disorganized visual presentation and detracts from the manuscript's professionalism. I recommend standardizing the legend placement for all figures (e.g., consistently in the upper-right corner or below the figure) while ensuring that legends do not obscure critical data.

Response to [R1-3]

We appreciate your insightful suggestions; we have carefully checked all figures to make our presentations consistent and follow professionalism.

In our revision, Figure 2 was not updated because they illustrate what we display to participants in our experiments, although legends are included in the graph main area. However, for other figures (Figs. 4, 5, 6, 7, 8, 9, and 11), we have modified them as consistent as possible and the python script to reproduce these figures was also included in our supporting files.

Our revision could not prevent readers from reading and understanding our findings.

Comment [R1-4]: Inconsistent Font Sizes Across Figures

The font sizes used in the figures vary significantly, with some being too small to read comfortably and others appearing disproportionately large. This lack of uniformity disrupts the visual coherence of the figures and hinders the reader's experience. I suggest adopting a consistent font size for all figure elements (e.g., axis labels, legends, and titles) that ensures legibility at standard page dimensions.

Response to [R1-4]

We thank for your comments. In our revised plotting script, attached in our supporting files, we have set all font sizes as consistent as possible using Matplotlib and parameters. We expect that our revised figures are more readable and consistent.

Section #3: Point-to-Point responses to Reviewer #2

General comment from Reviewer #2

I am pleased to confirm that this revised manuscript has now fully addressed the concerns from my prior review.

Response to Reviewer #2

Author response. We are happy to hearing from your confirmation on our revision conducted in PONE-D-25-17469R1. Again, we thank for the comments and suggestions from you in previous round, which contribute to improve our manuscript.

References

Masaki Suga, Yoshinori Nurata, and Kiyoaki Nakaminami. Experimental release of "japan map in multilingual notation" using vector tile data. *Bulletin of the Geospatial Information Authority of Japan*, 67, 2021.
