# Supplementary material for: User preferences in multi-objective routes: The role of gradient visualization and personality measures
Source: PLoS One. 2025 Aug 6;20(8):e0329387. doi: 10.1371/journal.pone.0329387 (PMC12327598; doi:10.1371/journal.pone.0329387)
Supplement: Attachment — Submitted filename: attachment.docx [file pone.0329387.s004.docx]

The manuscript presents a compelling and innovative exploration of user preferences in multi-objective route planning, effectively integrating gradient visualization and personality measures to advance personalized navigation design. However, it faces several challenges, including verbose and redundant language, vague or inconsistently defined terminology, insufficient quantitative rigor, unclear logical connections across sections, and inadequately designed figures that fail to meet scientific visualization standards. The following changes are recommended:

The abstract suffers from verbosity, vague terminology, and insufficient quantitative detail, reducing its conciseness and scientific rigor. The main problems are as follows:

- Verbose Language: Phrases like “we develop our framework to investigate” and repeated use of “our” (e.g., “our framework,” “our survey”) make the text less concise.
- Vague Terminology: Terms such as “multi-objective navigation,” “gradient information,” and “route-choice phenomena” are not clearly defined, potentially confusing readers.
- Lack of Specificity: Statements like “using modern multi-objective planning algorithms and crowdsourcing services” are overly broad, lacking details about the specific methods or technologies used.
- Insufficient Quantitative Detail: Claims such as “modestly encourages the selection of non-shortest and gentler routes” and “more pronounced preference” lack supporting data (e.g., percentages, statistical significance). The statement “route selection was also influenced by travel distance” is vague about the direction or magnitude of the effect.
- Unclear Relevance: Terms like “Big Five and Sensation Seeking dimensions” are standard in psychology but may confuse non-experts without context linking them to route choice.

The introduction provides a foundation for navigation research and research questions but is undermined by unclear content, loose structure, insufficient rigor, redundant language, imprecise terminology, and unclear positioning, reducing its academic persuasiveness and readability. The following changes are recommended:

- Overly Detailed Example: Example 1 includes excessive specifics (e.g., path numbers, colors), diverting focus from research objectives and failing to link clearly to the study’s goals.
- Weak Justification of Research Gap: The claim of “a significant gap” in understanding route evaluation lacks robust evidence, with only a few studies cited to support the link between personality traits and route choice, undermining the study’s necessity.
- Redundant and Subjective Language: Repeated use of “we” (e.g., “we develop a framework”) is overly subjective and detracts from the objective academic tone.
- Unclear Study Uniqueness: The introduction does not specify how the study addresses the understudied link between multi-objective routes and personality traits through methodological or theoretical innovation.

The related research section requires comprehensive refinement in content clarity, logical structure, scientific rigor, language expression, and terminology to enhance its academic value and readability. The following changes are recommended:

- Unclear Subsection Focus: Subsections (e.g., preparing navigation routes, personality in navigation, visualization and explanations) lack clear focus and logical connections, failing to align with the introduction’s research questions (RQ1-RQ3).
- Insufficient Scientific Rigor: Key claims lack literature support, and speculative statements (e.g., “we can hypothesize that walking time information would be valuable”) weaken credibility.
- Redundant and Inconsistent Language: Frequent use of phrases like “we can” or “for example” and inconsistent terminology (e.g., “personality measures” vs. other terms) reduce clarity.
- Vague Terminology: Terms like “crowdsourcing” and “multi-objective algorithms” are overly broad, lacking precise descriptions (e.g., “user-generated route data,” “Pareto-based route optimization”).

The results section requires comprehensive refinement in content clarity, analytical logic, scientific rigor, language expression, and data presentation to enhance its academic rigor and readability. The following changes are recommended:

- Weak Analytical Logic: The analysis does not clearly address research questions (RQ1-RQ3), relying heavily on data descriptions (e.g., means, statistical tests) without qualitative interpretation of trends or implications.
- Insufficient Scientific Rigor: Key statistical details (e.g., effect sizes, confidence intervals) are missing, and data filtering criteria (e.g., “valid participants”) are unclear.
- Unclear Personality Trait Analysis: The practical meaning of Sensation Seeking Scale (SSS) subdimensions (e.g., TAS, Dis) and their relevance to route choice are not explained beyond statistical differences.
- Redundant and Inconsistent Language: Phrases like “in the following, we...” are redundant, and terms like “gentler gradient” or “gradual route” are used inconsistently.
- Suboptimal Data Presentation: Direct references to figures (e.g., “Figure 4a shows...”) are overused, and figure annotations are not self-explanatory, limiting accessibility for readers without visuals.

The discussion is well-structured, but it lacks depth, specificity, and clarity in some areas, limiting its persuasiveness. The following changes are recommended:

- Superficial Result Interpretation: Responses to research questions (RQ1-RQ3) are clear but lack depth. For example, the conjecture about the lack of significant impact from gradient visualization (lines 406-407) lacks theoretical or data support.
- Brief Comparison to Prior Work: Comparisons with other studies (lines 423-441) are limited, mentioning only pedestrian-driver differences and personality measures, without highlighting the study’s unique contributions.
- Incomplete Limitations Analysis: Limitations (e.g., small sample size, suboptimal map visualization, regional focus, online survey constraints) are identified but not analyzed for their specific impacts on results (e.g., effect on statistical significance).
- Broad Future Directions: Suggestions like field experiments and expanding route attributes are vague, lacking specific experimental designs or methods.
- Verbose and Vague Language: Sentences like “we can start by clustering users according to these scores when recommending non-shortest routes” (line 421) are verbose and lack precision.

The figures are inadequately designed, overly casual, and fail to meet scientific visualization standards due to unreasonable layouts, non-standard plotting, and missing or unclear annotations. For example,

- Figure 1a: The legend obscures substantial content, reducing clarity.
- Figure 1b: Lacks a legend and uses non-standard English expressions.
- Figures 2a and 2b: The maps are not in standard English, but in Japanese, limiting accessibility.
- Figure 6d: Lacks a legend, reducing interpretability.

Similar problems exist in other diagrams and full verification is recommended.
