# Supplementary material for: User preferences in multi-objective routes: The role of gradient visualization and personality measures
Source: PLoS One. 2025 Aug 6;20(8):e0329387. doi: 10.1371/journal.pone.0329387 (PMC12327598; doi:10.1371/journal.pone.0329387)
Supplement: Attachment — Submitted filename: Reviewer Attachments.docx [file pone.0329387.s006.docx]

Thank you for submitting the revised manuscript. Upon reviewing the revised submission, I have identified several critical issues with the figures that significantly undermine the clarity of data presentation and the overall professionalism of the manuscript. Below, I outline these concerns and provide recommendations for improvement:

1. **Absence of Legend in Figure 9**
   Figure 9 lacks a legend, rendering it impossible for readers to accurately interpret the elements depicted. This omission severely compromises the figure’s readability and scientific rigor. I strongly recommend adding a clear and concise legend to Figure 9, ensuring that it corresponds directly to the figure’s content.
2. **Missing Units for Latitude and Longitude in Figure 10**
   The latitude and longitude axes in Figure 10 do not specify units (e.g., degrees, °), which violates standard scientific plotting conventions and risks ambiguity in data interpretation. I urge you to explicitly label the units for these axes and verify that similar issues do not affect other figures.
3. **Inconsistent Placement of Legends**
   The placement of legends across multiple figures appears arbitrary, with some positioned within the figure and others outside, in varying locations. This inconsistency creates a disorganized visual presentation and detracts from the manuscript’s professionalism. I recommend standardizing the legend placement for all figures (e.g., consistently in the upper-right corner or below the figure) while ensuring that legends do not obscure critical data.
4. **Inconsistent Font Sizes Across Figures**
   The font sizes used in the figures vary significantly, with some being too small to read comfortably and others appearing disproportionately large. This lack of uniformity disrupts the visual coherence of the figures and hinders the reader’s experience. I suggest adopting a consistent font size for all figure elements (e.g., axis labels, legends, and titles) that ensures legibility at standard page dimensions.

**Overall Recommendation**:
The issues outlined above indicate a systemic deficiency in figure design that requires comprehensive reformatting and optimization. I strongly advise consulting the target journal’s figure guidelines (e.g., PLOS ONE’s graphics requirements) to ensure compliance with academic publishing standards. Additionally, consider using professional plotting tools (e.g., Adobe Illustrator or Matplotlib in Python) to standardize and enhance the visual quality of the figures.
